# Objective versus subjective physical activity assessment in a clinical setting of patients with obesity and eating disorders: A concurrent validity study

**DOI:** 10.1007/s00508-026-02753-5

**Published:** 2026-05-12

**Authors:** Hans-Peter Wiesinger, Bettina Bannert, Elmar Kaiser, Patrick Kutschar

**Affiliations:** 1 Institute of General Practice, Family Medicine and Preventive Medicine, Center for Public Health and Healthcare Research, Paracelsus Medical Private University, Strubergasse 21, 5020 Salzburg, Austria; 2 Institute of Nursing Science and Practice, Center for Public Health and Healthcare Research, Paracelsus Medical Private University, Salzburg, Austria; 3 Department of Sport and Exercise Science, University of Salzburg, Salzburg, Austria; 4 Psychosomatic Clinic Eggenburg, Eggenburg, Austria

**Keywords:** Concurrent validity, Accelerometer-based multisensor device, IPAQ, Exercise therapy, Self-report bias

## Abstract

**Objective:**

Accurate assessment of physical activity supports a comprehensive understanding and treatment of obesity and eating disorders. Here, we assessed the concurrent validity of a questionnaire and accelerometry-based measurements in a clinical setting.

**Methods:**

From 2020 to 2022 a total of 23 patients from a psychosomatic clinic participated (21 with unspecified eating disorders, 2 with bulimia nervosa). During the 3rd to 4th week of the clinical stay patients completed the International Physical Activity Questionnaire-Short Form and wore an accelerometer-based multisensory device (SenseWear® Armband, SWA) for 7 days. Outcome measures included total physical activity (MET min·w^−1^) and sitting time (min·d^−1^). Concurrent validity (SWA as criterion) and proportional bias were assessed using regression analyses.

**Results:**

The questionnaire overestimated physical activity by ~40% (+394 MET min·week^−1^) and underestimated sitting time by ~30% (199 min·day^−1^) compared to the SWA. Within-patient correlation for physical activity was good (r = 0.88, *p* < 0.001) but impractical for sitting time (r = 0.41, *p* = 0.005). Greater inactivity led to greater reporting errors in sitting time (*p* = 0.005). Males underestimated sitting time more than females (*p* = 0.026). Converting objective to subjective measures resulted in typical errors of 238 MET min (total physical activity) and 88 min (sitting time).

**Conclusion:**

Objective accelerometer-based multisensory device monitoring should be incorporated into clinical settings to treat patients with obesity and eating disorders.

## Introduction

Accurate assessment of physical activity is essential for treating obesity and psychosomatic disorders, as clinical decision-making and evaluation of intervention effects rely on valid measures of activity [1]. However, self-reports often lack precision, particularly when recall is impaired by physical or psychological factors [2]. For patients with obesity and psychosomatic eating disorders, such recall impairments are likely further exacerbated by distorted activity perceptions and stress-related factors such as shame, stigmatization, and guilt [3]. Moreover, we expect the extent of inaccuracy in self-reported activity duration, frequency, or intensity to depend on the actual magnitude of activity. No previous study has examined the concurrent validity of subjective vs. objective physical activity measures and potential proportional bias [4] in this population and setting.

Doubly labelled water is the gold standard for measuring energy expenditure [5, 6] but impractical in routine clinical setting. Wearable accelerometer-based devices capture body movement rather than metabolic processes directly; nevertheless, they are widely accepted as viable, objective alternatives [6–8]. In contrast, self-report tools such as the International Physical Activity Questionnaire-Short Form (IPAQ) remain standard [8] despite known recall bias [9].

This reliance may stem from the acceptable reliability of IPAQ [10] and actigraphs [11] in healthy cohorts; however, even in these populations, within-subject correlations between these measures are impractical to very poor, ranging from r = −0.12 to r = 0.57 [10–12]. Self-report questionnaires, including the IPAQ, are known to systematically overestimate physical activity levels [8, 11, 13–16], likely due to inherent biases such as misinterpretation, recall, estimation and social desirability bias [3, 9, 17]. Validating these measures in clinical populations is critical for improving treatment, generating accurate research data, and providing clinicians with reliable, cost-effective tools.

We aimed to assess the concurrent validity of the IPAQ and accelerometer-based multisensor device (SWA) for (a) total weekly physical activity (MET·min·week⁻^1^) and (b) average daily sitting time (in minutes) in patients with obesity and eating disorders. The SWA served as the criterion measure. We hypothesized that the IPAQ would overestimate total physical activity and underestimate sitting time compared to actigraphy.

## Material and methods

### Study design

This concurrent validity study was conducted at the Psychosomatic Clinic Eggenburg (Austria) from May 2020 to March 2022. All patients provided written informed consent after receiving detailed information about the study procedures, with the option to withdraw at any time. Privacy and patient rights were protected throughout. All procedures were in accordance with the ethical standards of the responsible committee on human experimentation and with the Helsinki Declaration of 1975. Formal approval from an ethics committee was not obtained, as the study was classified as a quality assurance measure within routine clinical care (ClinicalTrials.gov identifier: NCT06784804).

### Patients

**Fig. 1:**
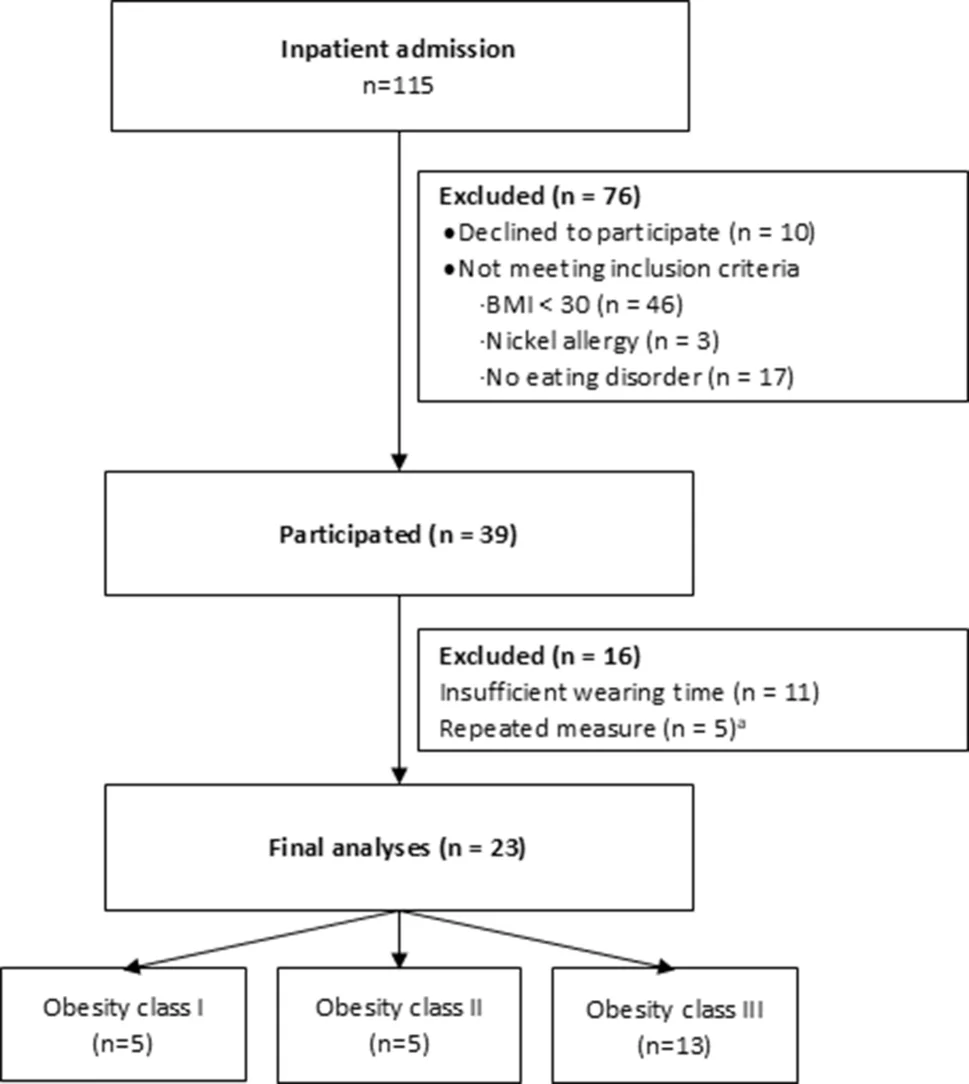
Flow chart of the study population selection process. *BMI* body mass index; Obesity classes I, II, III = BMI 30–34.9 kg∙m^−2^, 35–39.9 kg∙m^−2^, ≥ 40 kg∙m^−2^, respectively. ^a^Patients who were asked to re-wear the accelerometer and who were excluded from the analysis

Patient flow and characteristics are shown in Fig. 1 and Table 1. Eligibility criteria included: age ≥ 18 years, BMI ≥ 30 kg·m^−2^, sufficient German language proficiency, a clinical diagnosis of an eating disorder (ICD-10: F50.9, unspecified eating disorder; or F50.2 bulimia nervosa [18]), and a minimum clinic stay of 4 weeks. Participants also had to wear the SWA for ≥ 18 h per day, on at least 4 days, including one weekend day [19]. Exclusion criteria were inability to walk independently and nickel allergy. No interpolation or imputation of non-wear periods was performed; only recorded data were analyzed. Patients not meeting the valid SWA wear-time criteria were excluded.

**Table 1:** Patient characteristics

	Total	Obesity class I	Obesity class II	Obesity class III^b^	*p*-value
Sex (f/m)	23 (17/6)	5 (4/1)	5 (5/0)	13 (8/5)	–
Age (years)	42.1 ± 13.6	35.0 ± 12.6	38.4 ± 16.9	46.2 ± 12.1	0.241
BMI (kg·m^−2^)	40.5 ± 5.7	32.7 ± 1.2	38.4 ± 0.8*	44.4 ± 3.9***	< 0.001
Body fat (%)	43.5 ± 6.8	40.0 ± 5.6	46.7 ± 3.1	43.6 ± 7.8	0.302
Sleep efficiency (%)	81 ± 8	82 ± 8	84 ± 9	79 ± 8	0.503
SWA wearing time (min)	1400 ± 43	1427 ± 5	1361 ± 82*	1404 ± 16	0.042
Activity in min (> 3 MET)	94 ± 37	101 ± 40	96 ± 36	91 ± 39	0.865
Steps (*n*)	9935 ± 3050	10071 ± 3144	10778 ± 3455	9559 ± 3048	0.762
Clinical stay (days)	61 ± 16	60 ± 19	67 ± 11	59 ± 17	0.656
*Eating disorders*^a^					
Eating disorder (%)	91	60	100	100	–
Bulimia nervosa (%)	9	40	0	0	–
*Psychosomatic disorders*^a^					
Depression (%)	26	40	40	15	–
Personality disorders (%)	17	20	20	15	–
Neurotic disorders (%)	30	60	20	23	–
Bipolar disorders (%)	4	20	0	0	–

Values are mean ± SD, *BMI* body mass index; *MET* metabolic equivalent; *SWA* SenseWear accelerometer-based multisensor device; Obesity classes I, II, III = BMI 30–34.9, 35–39.9, ≥ 40 kg∙m^−2^

^a^All patients had a diagnosis of eating disorder, classified with the ICD-10 (F50.9 or F50.2 eating disorder) (Hilbert). Psychosomatic disorders were also diagnosed with the ICD-10 (F33, F60, F4, and F31 for depression, personality, neurotic and bipolar disorders, respectively)

^b^Two had a gastric bypass

^*^Significant difference to obesity class I (*p* < 0.05), *** highly significant differences to obesity class I and obesity class II (*p* < 0.001)

### Procedures and data collection

All patients received standardized instructions (investigator XX) on SWA use and IPAQ completion. During the first 2 weeks, patients followed standardized therapeutic routines, including walking, strength training, coordination exercises, and sports games. Body composition was measured in this period and SWA monitoring began on Monday of week 3 or 4 after patients’ clearance for supervised and independent exercise. Patients were instructed on SWA handling, including an activity log and completed the IPAQ for the same 7‑day period at the end of the week. The physical activity domains are based on the physical activity pyramid, with which patients were already familiar from clinical exercise education. Non-wear periods were logged, and participants were asked to maintain habitual activity levels during the monitoring phase. Due to the study design, blinding was not possible.

### Anthropometric measures and the International Classification of Diseases (ICD-10)

Standing height was measured using a wall-mounted stadiometer, and body mass was assessed barefoot in light clothing using a calibrated scale (Seca 635, Seca GmbH, Hamburg, Germany) at a similar time of day. The BMI was classified per WHO criteria [20]: obesity class I (30.0–34.9), class II (35.0–39.9), and class III (> 40 kg·m^−2^). Body fat percentage was measured via bioelectrical impedance analysis (Nutriguard M, Data Input GmbH, Darmstadt, Germany) before breakfast and without physical exercise the day before. Electrodes were placed on the dorsum of the foot and hand. Medical diagnoses and comorbidities were derived from routine clinical admission records. Eating disorders were classified according to ICD-10 (F50.0–F50.9), and psychosomatic comorbidities included depression (F33), personality disorders (F60), neurotic disorders (F4), and bipolar disorder (F31).

### Objective measure

The SenseWear® Armband Pro 3 (BodyMedia Inc., Pittsburgh, PA, USA) is a lightweight (83 g) multisensory device that combines a two-axis accelerometer with sensors for galvanic skin response, skin and ambient temperature, and heat flux. It is one of the most validated accelerometer-based multisensor devices, with concurrent validity established against doubly labelled water, showing an intraclass correlation coefficient (ICC_1.1_) of 0.80 [21]. The device was initialized using patient-specific data (sex, age, body mass, height) and worn on the dominant upper arm, positioned over the triceps midway between the acromion and olecranon. It was worn continuously except during personal hygiene. Data gaps were not imputed. Data were screened using InnerView software (BodyMedia Inc., Pittsburgh, PA, USA). Proprietary algorithms processed raw sensor data to estimate energy expenditure (kcal·min^−1^), metabolic equivalents (METs), step count, activity and rest duration, and sleep time. Activity intensity was categorized as low (< 3 METs), moderate (3–6 METs), or vigorous (≥ 6 METs) [22]. Inactive time was calculated as time spent ≤ 1.5 METs minus sleep duration. Time in bed and sleep duration were obtained from the device’s proprietary multisensor algorithm. Sleep efficiency was calculated by the SWA as the ratio of sleep duration to time in bed, multiplied by 100 (sleep duration/time in bed × 100).

### Subjective measure

The IPAQ is widely used to assess physical activity in individuals aged 15–69 years across diverse populations [5]. We used the short form of the IPAQ because it is the most common version in both clinical and research settings [23]. It includes 7 items covering the past 7 days. The IPAQ captures two intensity levels of physical activity (moderate and vigorous), identifies walking as a separate activity mode, and records sitting time as an indicator of sedentary behavior. The questionnaire assesses activity intensity, frequency, and duration, with test-retest reliability of r = 0.80 and criterion validity of r = 0.30 [10]. Total physical activity (MET min·week^−1^) was calculated using standardized MET values [22] by multiplying the frequency and duration of each activity type: 3.3 for walking, 4.0 for moderate, and 8.0 for vigorous activity. Weekly energy expenditure and fitness level estimates were derived accordingly [22]. Patients completed the IPAQ on Sunday evening, referencing the same 7‑day period during which the SWA was worn (Monday to the following Monday). Validity standards required data from at least three weekdays and one weekend day.

### Statistics

Data are shown as mean ± standard deviation (SD). Baseline differences across obesity classes were tested using a one-way ANOVA, with Tukey-corrected post hoc tests. Concurrent validity analyses followed Hopkins 2010 [24] using SWA as the criterion.

**Fig. 2:**
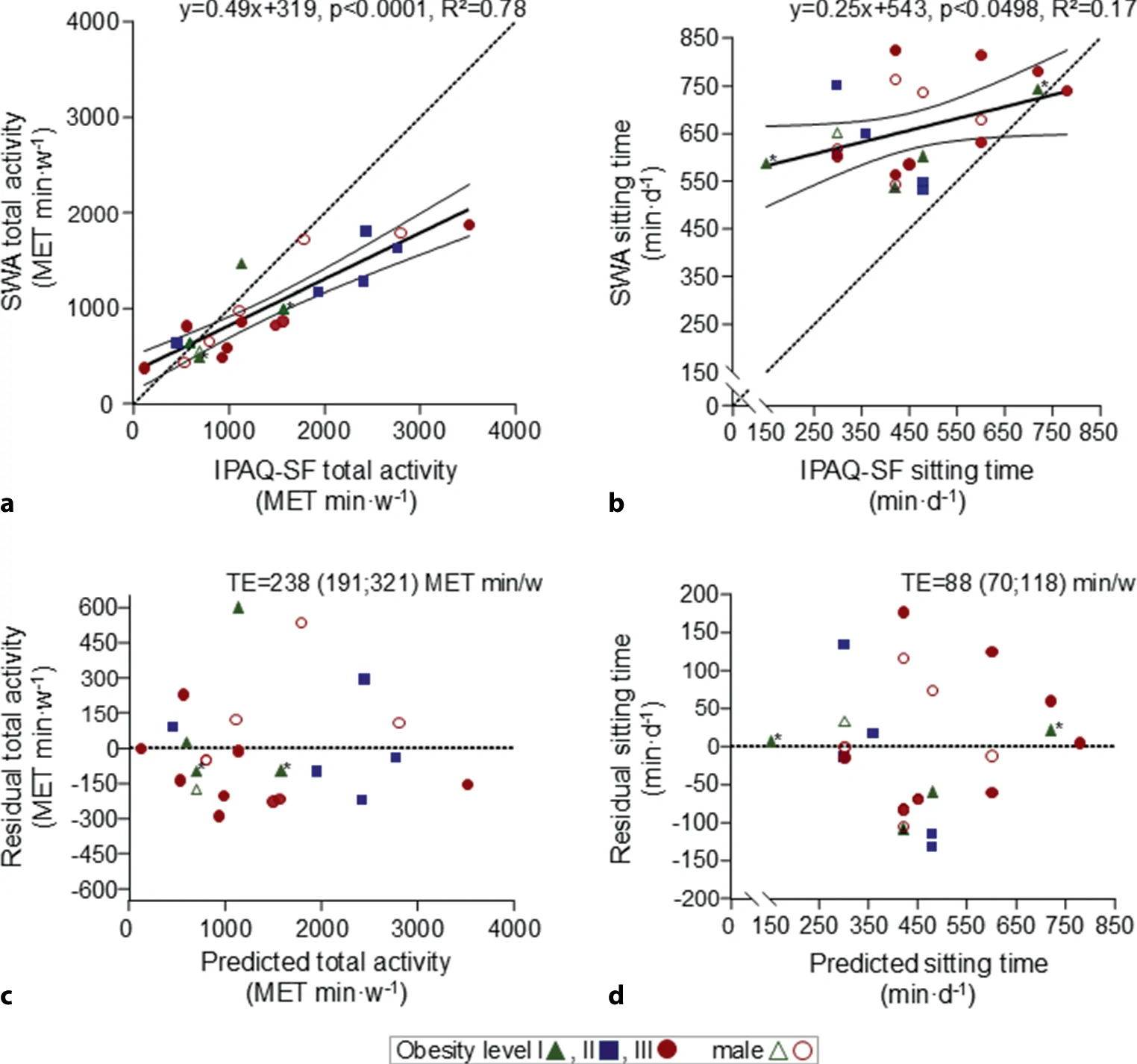
Regression analysis comparing SenseWear accelerometer-based multisensor device (SWA) and International Physical Activity Questionnaire-Short Form (IPAQ) measures for total physical activity and sitting time (**a**, **b**), along with residual versus predicted value plots (**c**, **d**) for patients with obesity and eating disorders. Solid lines represent the linear regression and its 95% confidence limits, while dashed lines denote the equality line (**a**, **b**). Residual plots (**c**, **d**) illustrate homoscedasticity, the typical error of the estimate (standard deviation of the residuals), and its 95% confidence limits for the residuals versus the predicted values. There were no outliers in either outcome measure (z-score ≤ 3). No outliers were identified in either outcome measure (z-score ≤ 3). Asterisk indicates the two patients with bulimia nervosa (ICD-10: F50.2); all other participants were classified as F50.9

Moderator effects of obesity classes (level III vs. I) and sex (male vs. female) were quantitatively tested, but separate regression analyses with these moderators were not justified due to the small number of patients. These analyses were exploratory and hypothesis-generating only; however, to gain an impression of the existence of subgroup-specific proportional biases, these moderators were visually incorporated into scatterplots (Fig. 2a, b) and their effects are discussed qualitatively. Scatterplots (Fig. 2a, b) and residuals versus predicted values (Fig. 2c, d) for total activity and sitting time were visually inspected for nonlinearity and nonuniformity. No variance stabilizing transformations (e.g., log transformation) were necessary. Pearson’s correlation coefficients were used to evaluate relationships between the objective (SWA) and subjective (IPAQ) measures. Correlation magnitudes were interpreted with strict concurrent validity criteria: r < 0.45, impractical; 0.45–0.70, very poor; 0.70–0.85, poor; 0.85–0.95, good; 0.95–0.99, very good and > 0.99, excellent [12]. Regression validity (proportional bias) was tested quantitatively by comparing linear regression lines with the line of equality [4]. The typical error (TE) was defined as the standard error of the estimate, calculated as the standard deviation of the residuals from a linear regression analysis (√[Σ(yᵢ − ŷᵢ)^2^/(n − 2)]), where yᵢ denote the observed value, ŷᵢ the predicted value, and n the sample size, representing the typical random deviation of individual estimates from the criterion measure. Paired sample Studentʼs t‑tests were used to compare the mean differences in the outcome measures between the two physical activity assessment tools. Percentage differences between SWA and IPAQ were calculated at the individual level using the formula: (IPAQ − SWA)/SWA × 100. The mean percentage difference and its 95% confidence interval were then derived from the individual values. Cohen’s *d* values of 0.2, 0.5, and 0.8 were interpreted as small, medium, and large effects, respectively. Analyses were conducted using SPSS V.29.0 (SPSS Inc., Chicago, IL, USA). Figure 2 was created with GraphPad Prism 9.1.2 (GraphPad Software Inc., San Diego, CA, USA). The significance level was set at *p* < 0.05 (two-sided).

## Results

During the study period, ~43% of all patients admitted to the psychosomatic clinic met the inclusion criteria, with ~34% participating (80% participation rate among eligible patients). Ultimately, ~20% of all patients were included in the final analysis, while ~14% were excluded due to improper SWA usage (Fig. 1). Patient characteristics were generally similar across obesity classes I–III, except for total SWA wear time and, naturally, BMI. Of the patients 13 suffered from psychosomatic disorders (Table 1).

Table 2 summarizes SWA and IPAQ measures of physical activity and sitting time, as well as the potential influence of obesity severity and sex. On average, patient self-reports (IPAQ) overestimated total physical activity by ~34% (*p* = 0.001) and underestimated sitting time by ~30% (*p* < 0.001). Hence, total activity was 394 MET min·week^−1^ higher, while sitting time was 199 min·day^−1^ lower in self-reported compared to SWA measures. These discrepancies between subjective and objective measures were also observed across all obesity classes and both sexes, with significant differences for most comparisons, except for total activity in males (*n* = 6) and obesity class I (*n* = 5). A moderator effect of sex was found for sitting time (*p* = 0.026), showing that male self-reports deviated more from SWA measures than females (~18%, 128 min·day^−1^). Physical activity discrepancies were ~29% (moderator effect of 262 MET min·week^−1^) higher in females than in males, but the difference was not statistically significant (*p* = 0.282).

**Table 2:** Comparison of total activity and sitting time

	Range	Mean ± SD	Difference (%) Mean; 95% CI	Cohen’s d
***Total activity (MET min· week***^***−1***^***)***				
*SWA*	375–1883	998 ± 495	–	–
*IPAQ*	120–3519	1392 ± 895	34; 15–53**	0.77
*1*				
Obesity I (SWA)	485–1473	829 ± 409	–	–
Obesity I (IPAQ)	594–1575	938 ± 413	19; −23 to 61	0.32
Obesity II (SWA)	632–1802	1301 ± 453	–	–
Obesity II (IPAQ)	462–2772	2010 ± 913	47; −10 to 104*	0.32
Obesity III (SWA)	375–1883	946 ± 520	–	–
Obesity III (IPAQ)	120–3519	1330 ± 940	35; 5–64*	0.73
Females (SWA)	440–1883	1053 ± 491	–	–
Females (IPAQ)	462–3519	1516 ± 877	41; 21–62**	0.88
Males (SWA)	375–1792	841 ± 516	–	–
Males (IPAQ)	120–2799	1042 ± 927	13; −43 to 68	0.43
**Moderator effects (∆ IPAQ-SWA)**				
Obesity III–Obesity I	–	276 ± n. a.	15; −28 to 59	0.57
Males–females	–	−262 ± n. a.	−29; −20 to 78	0.51
***Sitting time (min·week***^***−1***^***)***				
*SWA*	530–824	656 ± 94	–	–
*IPAQ*	150–780	457 ± 157	−30; −40 to −21***	−1.37
Obesity I (SWA)	539–743	625 ± 77	–	–
Obesity I (IPAQ)	150–720	414 ± 213	−35; −71 to 1	−0.32
Obesity II (SWA)	530–751	616 ± 89	–	–
Obesity II (IPAQ)	300–480	384 ± 91	−35; −46 to −24*	−1.36
Obesity III (SWA)	542–824	684 ± 99	–	–
Obesity III (IPAQ)	300–780	501 ± 149	−27; −38 to −16***	−1.36
Females (SWA)	530–816	651 ± 97	–	–
Females (IPAQ)	150–780	485 ± 168	−26; −37 to −14***	1.12
Males (SWA)	586–824	670 ± 93	–	–
Males (IPAQ)	300–480	375 ± 84	−44; −56 to −31**	3.2
**Moderator effects (∆ IPAQ-SWA)**				
Obesity III–Obesity I	–	28 ± n. a.	8; −21 to 37	0.19
Males–females	–	128 ± n. a.	−18; −33 to −3*	0.94

Values are mean ± SD standard deviation; Obesity classes I, II, III = BMI 30–34.9, 35–39.9, ≥ 40 kg∙m^−2^

*BMI* body mass index, *CI* confidence interval, *MET* metabolic equivalent, *SWA* SenseWear accelerometer-based multisensor device, *IPAQ* International Physical Activity Questionnaire-Short Form, **∆** difference, * *p* < 0.05, ** *p* < 0.01, *** *p* < 0.001

There was a good within-patient correlation for physical activity measures (r = 0.88, 95% CI: 0.77–0.94, *p* < 0.001). In contrast, the within-patient correlation for total sitting time was impractical (r = 0.41, 95% CI: 0.07–0.67, *p* = 0.050). Linear regressions for both outcome measures were significant (*p* ≤ 0.05). Measures of differences between the regression line and the line of identity showed a trend for a proportional bias for physical activity (t = 1.80, *p* = 0.080) and a significant proportional bias for sitting time (t = 2.97, *p* = 0.005). Hence, when subjective IPAQ measures were converted to the objective SWA measures, the typical errors of the estimates were substantial, with 238 MET min·week^−1^ for total activity and 88 min·day^−1^ for sitting time. For total activity, positive linear trends with comparable proportional bias across obesity classes I–III and sex are apparent (Fig. 2a); however, for sitting time, a negative slope is observed for patients with lower obesity classes (I and II), and possibly also for male patients, while the overall slope is positive (r = 0.42).

## Discussion

This is the first study to compare IPAQ and SWA-based assessment in patients with obesity and comorbid eating disorders. Our results showed that the IPAQ substantially overestimated total physical activity while underestimating total sitting time. This bias was consistent across obesity classes I–III but males showed a greater underestimation of sitting time than females. Proportional bias analyses revealed that individuals with higher activity levels tended to have greater subjective overestimations of physical activity, whereas those with lower sedentary levels underestimated their sitting time more. These findings highlight the need for objective measures like SWA to assess physical activity in this population and setting.

The observed overestimation and underestimation align with our hypothesis, previous findings in both healthy individuals [8, 15] and other clinical populations [11, 13, 14]; however, the mean discrepancy for total physical activity in our cohort was smaller than in studies of healthy individuals, where differences of ~1500 MET min·week^−1^ were reported [7]. This observed difference likely reflects the proportional bias in our data. For example, at the average activity level of 3765 MET min·week^−1^ reported in healthy individuals [8], our regression model would predict a similar discrepancy of ~1500 MET min·week^−1^. The underestimation of sitting time by more than 3 h·day⁻^1^ mirrors findings in patients with upper body orthopedic injuries [11], and only 1 of 23 patients in our study reported less sitting time via IPAQ than recorded by SWA, emphasizing the systematic nature of this bias; however, clinicians, researchers, and other practitioners should be aware of the bidirectional pattern of proportional bias. Thus, reported overestimations or underestimations of physical activity and sitting time can be reversed in individuals with very low activity levels (low MET values and high sitting time). Indeed, our data indicated that this was the case for a few individuals with very low activity levels in our setting.

For subgroup effects, previous studies have consistently shown that self-report bias when using the IPAQ occurs across sexes [8], BMI levels [8], and age groups [25–27], but generally without clear and systematic differences between these subgroups [8, 13]. Accordingly, we did not include age as a moderator; however, we anticipated effects of BMI and sex [3], especially given the awareness of the role of physical activity in treatment and that those with severe obesity may even suffer from a greater social desirability bias. Sex was included because body image stigma can differ [3] and women often show greater social desirability bias in diet and weight loss reports [25]. In this context, observed sex difference, with males reporting greater discrepancies in sitting time compared to objective SWA measures, was unexpected, especially given that recommended activities during clinical stays did not differ by sex and an opposite trend was found for physical activity. Although we could not identify a plausible explanation for this sex effect, it suggests that sex-specific bias can vary by outcome measure. Future studies with larger cohorts are needed to explore such subgroup effects more precisely.

Furthermore, a key distinction of our study is the inclusion of patients with comorbid eating and psychosomatic disorders. Such conditions may impair cognition, memory recall and motivation, potentially affecting the accuracy of IPAQ responses. For example, patients with bipolar disorder may overestimate physical activity during manic phases and underestimate it during depressive phases [28]. In addition, disturbances in body image and weight-related self-perception, which are common in patients with obesity and eating disorders, may influence how individuals evaluate and report their own physical activity levels. Perceived rather than actual activity may therefore shape questionnaire responses. Although subgroup prevalence of these conditions was balanced, they likely contributed to lower within-subject correlations and higher random error (Fig. 2c, d). As psychosomatic conditions are more prevalent among obese individuals [29], excluding such patients would not reflect real-world clinical settings. Therefore, we recommend that future studies should also assess cognitive function as an additional moderator beyond sex, BMI and mental health status. As suggested decades ago by Durante and Ainsworth [3] and echoed by recent studies [26, 27], incorporating cognitive capacity or educational attainment as a proxy [14, 25] into survey designs could improve data quality.

Cognitive models from psychological and survey research frameworks [3] may provide valuable insights into the biases observed in self-reports. Standardized questionnaires, such as the IPAQ, impose substantial cognitive demands, requiring participants to accurately recall, quantify, and report the duration and intensity of their physical activities. These tasks may be particularly challenging for obese patients facing stress or stigma from psychosomatic disorders. To improve data quality, we recommend experimental studies that compare the current IPAQ format with less cognitively demanding alternatives, such as questions with predefined response categories, to better accommodate these challenges.

To our knowledge, this is the first study to quantitatively assess proportional bias between IPAQ and SWA data. We found that patients with higher physical activity levels are more likely to overestimate their exercise time, while those with lower sedentary levels underestimate their sitting time. This aligns with qualitative observations of Fig. 1 in Johannsen et al. [21] and Chastin et al. [30]. While obesity class and sex did not moderate total activity discrepancies (Fig. 2a), they appeared to influence sitting time estimates (Fig. 2b): patients with lower BMI and males reported lower sitting time relative to SWA data. This points to the need for adequately powered subgroup analyses and bias correction models to adjust IPAQ data for objective use.

From a broader perspective, the discrepancies between subjective and objective methods may extend to other phenomena assessed via standardized tools in patients with obesity. While objective tools like SWA are feasible in clinical settings, they are less applicable for use at home or after hospitalization. Hybrid methods that combine subjective reports with objective data, while accounting for factors like sex, cognition, and mental health, could improve accuracy [2, 8]. Interventions should also address social desirability and stigma, tailoring approaches by gender and psychological profile to enhance self-report validity. Yet, in summary, the large discrepancies between SWA and IPAQ measures of physical activity and sitting time clearly suggest that accelerometer-based multisensor device or other objective measures should be implemented in such settings whenever possible to accurately assess intervention effects.

## Strengths and limitations

The main findings stand robust, supported by well-accepted objective (SWA) and subjective (IPAQ) measures, rigorous interpretation of within-subject correlation, and an 80% participation rate representative of the study population. If questionnaire-based assessments continue to be used in clinical or large-scale epidemiological settings where objective monitoring is not feasible, adequately powered studies incorporating sociodemographic, cognitive, and personality factors (e.g., social desirability) are warranted to better quantify and potentially correct subgroup-specific biases. The 7‑day recall format of the IPAQ may have introduced recall bias in this psychosomatic clinical population. Objective monitoring remains the preferred method for physical activity assessment in this setting, while structured daily diaries or adjunct use of devices such as the SenseWear Armband may be considered when continuous objective measurement is not feasible. The SWA’s upper arm placement, while manufacturer-recommended, may miss activities with limited arm movement (e.g., cycling), potentially increasing the discrepancy between IPAQ and actual activity. Despite being among the best devices, newer accelerometer-based multisensor devices may offer slightly improved accuracy. Furthermore, although both IPAQ and SWA outputs are expressed in MET units, these values are derived from movement-based activity or self-reported behavior rather than from direct metabolic measurements. Consequently, comparisons should not be interpreted as concurrent validity of operational proxies of physical activity, rather than as equivalence to doubly labelled water-based measurements of energy expenditure. Because resting oxygen consumption per kilogram body mass may differ in individuals with obesity, absolute MET values should be interpreted cautiously. Accordingly, our conclusions focus on relative discrepancies between assessment methods rather than on absolute activity magnitudes.

## Data Availability

The original contributions presented in the study are included in the article; further inquiries can be directed to hans-peter.wiesinger@pmu.ac.at.

## Abbreviations

BMI: Body mass index
ICD: International Classification of Diseases
IPAQ: International Physical Activity Questionnaire—Short Form
SWA: SenseWear accelerometer-based multisensor device
